# Peripheral Vascular Access in Infants: Is Ultrasound-Guided Cannulation More Effective than the Conventional Approach? A Systematic Review

**DOI:** 10.3390/medicina61081321

**Published:** 2025-07-22

**Authors:** Cristina Casal-Guisande, Esperanza López-Domene, Silvia Fernández-Antorrena, Alberto Fernández-García, María Torres-Durán, Manuel Casal-Guisande, Alberto Fernández-Villar

**Affiliations:** 1 Emergency Department, Hospital Universitario Rey Juan Carlos, 28933 Móstoles, Spain; cristinacasalguisande@gmail.com; 2 Faculty of Health Sciences, University Alfonso X el Sabio, 28691 Villanueva de la Cañada, Spain; edomelop@uax.es; 3 Internal Medicine Department, Hospital Universitario Álvaro Cunqueiro, 36312 Vigo, Spain; silvia.fernandez.antorrena@sergas.es; 4 Diagnostic Imaging Department, Hospital Ribera Povisa, 36211 Vigo, Spain; alberto.fernandez.garcia@outlook.es; 5 Pulmonary Department, Hospital Álvaro Cunqueiro, 36312 Vigo, Spain; maria.luisa.torres.duran@sergas.es (M.T.-D.); jose.alberto.fernandez.villar@sergas.es (A.F.-V.); 6 NeumoVigo I+i Research Group, Galicia Sur Health Research Institute (IIS Galicia Sur), SERGAS-UVIGO, 36312 Vigo, Spain; 7 Centro de Investigación Biomédica en Red, CIBERES ISCIII, 28029 Madrid, Spain; 8 Fundación Pública Galega de Investigación Biomédica Galicia Sur, Hospital Álvaro Cunqueiro, 36312 Vigo, Spain; 9 School of Industrial Engineering, University of Vigo, 36310 Vigo, Spain

**Keywords:** ultrasound-guided vascular cannulation, peripheral vascular access, infants, pediatric emergencies, nursing practice, systematic review

## Abstract

*Background and Objectives*: Peripheral vascular access in infants is a frequent but technically challenging procedure due to the anatomical characteristics of this population. Repeated failed attempts may increase complications and emotional stress for both patients and healthcare professionals. This systematic review aimed to evaluate the efficacy and safety of ultrasound-guided peripheral vascular cannulation compared to the conventional or “blind” technique in infants. *Materials and Methods*: A systematic review was conducted in accordance with PRISMA guidelines. The PubMed database was searched for studies published between 2017 and 2025. Studies comparing both techniques in infants under two years of age were selected, evaluating variables such as the number of punctures, first-attempt success, healthcare staff perception, associated stress, and the role of simulation in training. *Results*: Eleven studies were included, comprising clinical trials, observational studies, and training program assessments from different countries. Most reported a higher first-attempt success rate with the ultrasound-guided technique (often exceeding 85%), along with fewer punctures and complications, particularly among less-experienced professionals. Improvements in staff perception were also observed following structured training. The impact on stress experienced by patients and families was less frequently assessed directly, although some studies reported indirect benefits. *Conclusions*: Ultrasound-guided peripheral vascular cannulation appears to be more effective and safer than the conventional technique in infants, particularly in complex or critical care contexts. Its implementation requires specific training and appropriate resources but could significantly improve clinical outcomes and the pediatric patient experience.

## 1. Introduction

Peripheral vascular access (PVA) is one of the most common clinical procedures in hospital settings, used for the administration of fluids and medications through the bloodstream [1,2]. Although highly standardized in adults and typically performed by nursing staff, PVA presents multiple technical challenges in pediatric patients—particularly infants—that compromise its effectiveness [3].

These challenges are mainly due to the physiological and anatomical characteristics of infants, such as limited vascular access and a higher proportion of subcutaneous tissue. These factors significantly hinder vein visualization, palpation, and depth estimation, increasing the risk of failure [4,5]. As a result, the conventional or “blind” technique has a higher error rate in this population, often leading to more puncture attempts, longer procedure times, and increased stress for both patients, families, and healthcare professionals [4,6].

Various types of peripheral insertion catheters have been developed to suit different clinical needs, including short peripheral intravenous catheters (PIVCs), midline catheters, and peripherally inserted central catheters (PICCs), each with specific indications, benefits, and limitations [7,8]. Device selection should be based on treatment duration, patient characteristics, and the nature of the substance to be administered, as inappropriate choices may compromise efficacy and increase complication risk [9,10,11].

When PVA fails, alternatives such as intra-osseous cannulation may be considered, though this option also carries significant risks and long-term complications in pediatric patients, such as extravasation, osteomyelitis, or compartment syndrome [12,13,14,15,16].

In recent years, ultrasound-guided cannulation has gained growing importance as a complement to conventional approaches based on palpation or visual inspection. Its ability to provide real-time visualization of vascular structures has been shown to improve first-attempt success rates, reduce the number of punctures required, shorten procedure duration, and decrease the incidence of associated complications [17,18,19,20]. These advantages have been particularly documented in patients with difficult vascular access, including critically ill pediatric patients [20]. However, its widespread implementation still faces barriers, including the need for specific training. Training nursing staff in this technique is essential, and clinical simulation—including virtual reality—has proven effective for skill acquisition in a controlled environment [21,22,23,24,25,26,27,28,29,30,31,32,33].

Despite the growing number of publications on ultrasound-guided vascular cannulation, questions remain regarding its applicability, effectiveness, and efficiency in infants, especially in emergency and intensive care settings. Therefore, this systematic review aims to evaluate the efficacy of the ultrasound-guided technique versus the conventional or “blind” technique for peripheral vascular access in infants, considering indicators such as the number of punctures, the stress generated, healthcare staff perceptions of its usefulness, and the importance of simulation in training.

## 2. Materials and Methods

### 2.1. Study Design

A systematic review of the scientific literature was conducted with the aim of evaluating the efficacy of ultrasound-guided peripheral vascular cannulation in infants, compared to the conventional or “blind” technique, within pediatric emergency and intensive care settings.

This review was designed and carried out in accordance with the recommendations of the PRISMA statement (Preferred Reporting Items for Systematic Reviews and Meta-Analyses) [34], ensuring a transparent, rigorous, and reproducible process.

### 2.2. Research Question

Could the use of ultrasound-guided peripheral vascular cannulation in infants treated in pediatric emergency and intensive care units reduce the number of punctures and the level of stress in patients, their families, and healthcare professionals compared to the conventional technique?

### 2.3. Inclusion and Exclusion Criteria

Studies were included if they met the following criteria: publications from 2017 to 2025, in Spanish, English, or Portuguese; free full-text availability; studies conducted on infant populations analyzing peripheral vascular cannulation using an ultrasound-guided technique; inclusion of direct comparisons with the conventional technique; and evaluation of variables such as number of punctures, stress perceived by the patient or their caregivers, nursing staff perception or experience, and the role of simulation in training.

Studies were excluded if they did not explicitly refer to the use of the ultrasound-guided technique, did not present comparisons between techniques, did not provide objective data on the efficacy or impact of the procedure, if they were conducted on animal models, simulations without direct clinical application, or were not available in full text.

### 2.4. Literature Search Strategy

The search was conducted between November 2024 and April 2025 in the PubMed database. Controlled descriptors (MeSH terms) and free-text keywords were used, combined with the Boolean operators AND and OR, adjusting the strategies to the specific characteristics of the database. To complement the search, the references of the selected articles were manually reviewed.

The following search strategies were used:

(“Catheters”[MeSH] OR “peripheral venous catheter”) AND (“Pediatrics”[MeSH] OR “Infant”[MeSH]) AND (“Stress”[MeSH] OR “Complications”); (“Peripheral venous catheter” OR “vascular access” OR “intravenous access”) AND (“complications” OR “techniques” OR “ultrasound” OR “training”).

The results were managed manually to remove duplicates. Study selection was performed in two phases—first by screening titles and abstracts, and subsequently through full-text reading. This process was carried out by two independent reviewers (C.C.-G. and E.L.-D.); in the case of a disagreement regarding a study’s eligibility, a third reviewer (A.F.-G.) was consulted to resolve the discrepancy by consensus.

### 2.5. Synthesis of Results

The data extracted from the selected studies were organized into a summary table that includes their main methodological characteristics and relevant findings. The aspects analyzed included the number of cannulation attempts, procedure duration, presence of complications, stress perceived by patients and families, and the nursing staff’s assessment of the technique used.

A meta-analysis was not conducted due to the heterogeneity of study methodologies, designs, and outcome variables. For this reason, the findings have been synthesized descriptively and in a structured manner, allowing for a qualitative interpretation of the available data.

## 3. Results

### 3.1. Study Selection

The initial literature search identified a total of 147 records through PubMed, with 5 additional records being obtained by reviewing references.

Of these, 60 full-text articles were assessed. After applying the inclusion criteria, a total of 11 studies were included in the systematic review.

**Figure 1:**
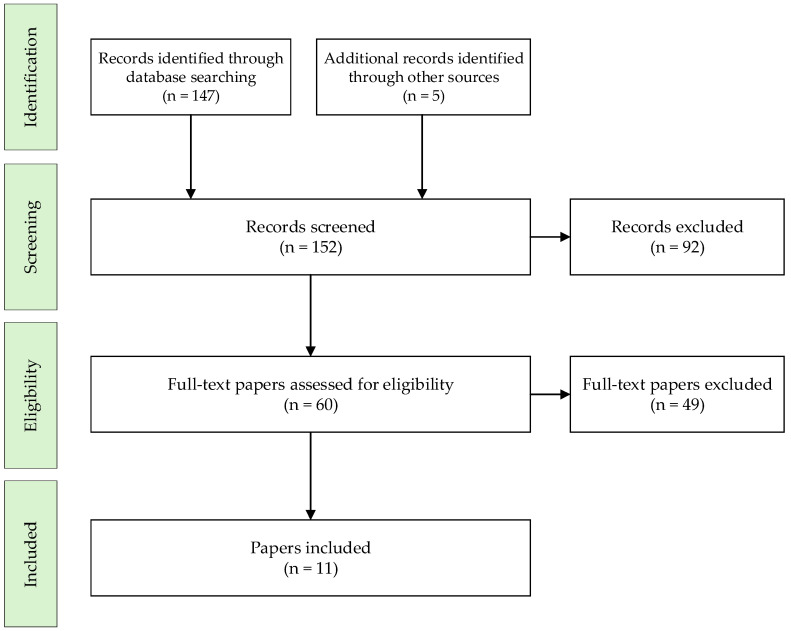
PRISMA flow diagram of the study selection process.

The selection process is illustrated in Figure 1 using the adapted PRISMA diagram.

### 3.2. Characteristics of the Included Studies

The 11 studies included were published between 2019 and 2024 and were conducted in various pediatric settings in countries such as Spain, Japan, Australia, Austria, Denmark, the United Kingdom, and the United States. The methodological designs included clinical trials, observational studies, and systematically evaluated training programs, with varying sample sizes, primarily involving infants under 2 years of age.

In most studies, ultrasound-guided peripheral vascular cannulation was compared to the conventional technique. The most commonly analyzed variables were the number of attempts, first-attempt success rate, procedure duration, healthcare staff perception, and stress experienced by patients or their families. The methodological characteristics of the included studies are presented in Table 1.

### 3.3. Main Results

The results of the selected studies were grouped into the following four thematic areas: (a) stress reduction, (b) reduction in the number of punctures, (c) nursing staff perception, and (d) training and education. The main findings organized by these thematic areas are summarized in Table 2.

The findings presented reflect a consistent trend in favor of using the ultrasound-guided technique for peripheral vascular cannulation in pediatric populations. Regarding perceived stress, although few studies evaluated it directly, some authors noted that factors such as fewer attempts, shorter procedure duration, and greater perceived safety may help reduce anxiety in patients and their families. The study by Castillo and Corral [35] particularly highlights the role of local anesthesia in the pain experience, without observing significant differences attributable to the use of ultrasound guidance itself.

In terms of clinical efficacy, the results are robust. Studies such as those by Takeshita et al. [36], Oulego-Erroz et al. [37], and Kleidon et al. [38] showed clearly higher first-attempt success rates with the ultrasound-guided technique, even more so when the operator had less experience or the patient’s anatomy posed greater challenges. These benefits also translated into lower complication rates and less need for redirection or catheter replacement, reinforcing the value of the technique in highly vulnerable settings.

Nursing staff perception was evaluated in two studies [30,31], where widespread acceptance of the technique was observed, especially after structured training. In this regard, the clinical utility, visual precision, and increased sense of control during the procedure were positively assessed. High scores in fidelity and satisfaction tools further support the feasibility of incorporating this approach routinely into clinical practice.

Finally, studies focused on training and education [32,33,41] demonstrated the impact of training programs on improving competencies. Both conventional training and those incorporating simulation or virtual reality significantly contributed to increasing success rates, professional confidence, and procedural safety. These findings highlight the importance of formal and continuous education as a foundation for the effective and safe implementation of the technique in pediatric clinical settings.

## 4. Discussion

### 4.1. Clinical Efficacy and Procedural Outcomes

The findings of this systematic review reinforce the clinical utility of ultrasound-guided peripheral vascular cannulation in pediatric populations, especially in infants. The analyzed studies consistently indicate that this technique significantly improves first-attempt success rates, reduces the number of required punctures, and shortens procedure duration compared to the conventional or “blind” technique. These benefits are particularly relevant in critical settings such as emergency departments or pediatric intensive care units (PICUs), where efficacy and speed are essential for patient safety.

The complexity of vascular access in infants is due to anatomical factors such as a smaller vessel diameter, a greater proportion of subcutaneous tissue, and patient mobility, all of which hinder the identification of palpable or visible veins. In this context, real-time ultrasound guidance facilitates vessel localization and improves puncture precision, reducing failed attempts and associated complications. This positive effect was demonstrated in studies by Takeshita et al. [36], Kleidon et al. [38], Gopalasingam et al. [40], and Triffterer et al. [39], which reported first-attempt success rates above 85% with ultrasound guidance, compared to rates below 60% with the conventional technique.

Regarding the time required to complete cannulation, the results were more heterogeneous. Some studies, such as that by Takeshita et al. [36], showed a significant time reduction in the ultrasound-guided group. However, others—like Gopalasingam et al. [40]—did not observe differences in total time, though they did report fewer needle redirections and punctures. These findings suggest that the operator’s experience with ultrasound is a key factor. The study by Oulego-Erroz et al. [37] confirmed that the benefits of ultrasound were more pronounced among less experienced PICU professionals, reinforcing the need for technical training to optimize outcomes.

### 4.2. Technical Approaches, Complications, and Patient Selection

Ultrasound-guided peripheral vascular cannulation can be performed using two primary imaging approaches [42]—the transverse (out-of-plane) technique and the longitudinal (in-plane) technique. Each method offers distinct advantages and limitations [43,44]. The in-plane approach allows for the continuous visualization of the entire needle shaft and tip during advancement, which may enhance accuracy and reduce the risk of posterior wall puncture. Conversely, the out-of-plane approach provides a clearer cross-sectional view of the vessel and can be easier to learn, but it requires more skill to track the needle tip accurately in real time. Although both techniques are used in pediatric practice, none of the studies included in this review reported outcomes stratified by the approach used. Future research should consider this technical variable when evaluating ultrasound-guided cannulation in infants, as the choice of technique may influence success rates and safety depending on operator experience and patient characteristics.

Although most of the studies included in this review reported lower complication rates with ultrasound-guided cannulation compared to the conventional method, potential adverse events should still be considered. Complications such as hematoma, phlebitis, infiltration, or extravasation remain possible, especially in cases involving less experienced operators or infants with particularly challenging vascular access [45]. While ultrasound guidance enhances accuracy and procedural success, it does not completely eliminate these risks. Therefore, the effectiveness of the technique depends not only on the availability of appropriate equipment but also on continuous and structured training to ensure the competence of the healthcare professionals performing the procedure.

Ultrasound-guided cannulation appears particularly beneficial for specific infant subgroups, including those with obesity, edema, dehydration, or chronic conditions requiring frequent access. Preterm and low-birth-weight infants, who typically have fragile and poorly visible veins, also represent a high-benefit group. Additionally, critically ill infants in emergency or intensive care settings—where rapid and reliable vascular access is crucial—are particularly suitable candidates. In such scenarios, ultrasound guidance may also compensate for operator inexperience, further enhancing its clinical utility.

### 4.3. Clinical Judgment and Decision-Making Criteria

Although specific pediatric guidelines remain limited, current evidence supports several clinical indications for ultrasound-guided peripheral vascular access in infants. These include cases with known or suspected difficult access, multiple prior failed attempts, or an urgent need for rapid and reliable cannulation. Conversely, in infants with clearly visible or palpable veins, the conventional technique may suffice and offer greater time and cost efficiency. Relative contraindications include the lack of operator training or absence of suitable ultrasound equipment. Therefore, the decision should balance patient-specific needs with institutional resources, aiming to maximize safety and procedural success.

However, the widespread implementation of ultrasound-guided cannulation is hindered by the absence of standardized clinical criteria and validated decision-making tools. In current pediatric practice, the choice to use ultrasound often relies on individual clinical judgment or vague descriptors such as “difficult vascular access,” which lack operational definitions. This is particularly problematic in time-sensitive environments like emergency departments.

To address this gap, future research should focus on the development and validation of pediatric-specific assessment tools—such as scoring systems based on patient characteristics and prior failed attempts—that can guide a more equitable, efficient, and evidence-based application of this technique.

### 4.4. Emotional Impact and Staff Perceptions

In terms of emotional impact, the study by Castillo and Corral [35] explored the relationship between ultrasound use and procedural stress, concluding that no significant differences were observed per se. Rather, stress reduction appeared more closely linked to the use of anesthetics. Although some studies suggest that ultrasound-guided peripheral cannulation may alleviate stress for pediatric patients and their families, the current evidence is insufficient to isolate the effect of ultrasound itself. A major limitation is the inconsistent reporting of analgesia or anesthesia use across studies. Notably, only Castillo and Corral [35] explicitly examined this variable. Therefore, it remains unclear to what extent the emotional benefit is attributable to ultrasound guidance alone. Future research should include the standardized control of these factors to clarify their respective contributions within the broader framework of patient-centered care.

On the other hand, healthcare staff perception was broadly positive. Studies such as those by Carrie Ng et al. [30] and López-Álvarez et al. [31] revealed improved nursing staff attitudes following theoretical and practical training sessions, with particular appreciation for the technique’s usefulness and ease of learning. The implementation of structured training programs, as described by McKinney et al. [32] and Hackett et al. [33], has been shown to not only boost staff confidence but also enhance clinical success rates in real-life cannulations. Similarly, Andersen et al. [41] demonstrated that combining ultrasound training with virtual reality improves technical performance in students without prior experience.

### 4.5. Costs and Technological Alternatives

One of the most frequently cited barriers to the widespread adoption of ultrasound-guided peripheral vascular cannulation in pediatric care is the cost associated with acquiring, maintaining, and updating ultrasound equipment, as well as the resources required for adequate staff training. These limitations can be particularly pronounced in low-resource or rural healthcare settings. Nevertheless, several approaches may help to overcome these challenges. The increasing availability of compact and portable ultrasound devices at lower cost offers a promising avenue to facilitate broader access, especially when integrated into point-of-care workflows. Furthermore, establishing centralized or shared training programs—possibly supported by regional health authorities or academic institutions—can reduce the financial burden on individual hospitals. Incorporating simulation-based education and e-learning modules can also promote efficient and scalable training without the need for continuous on-site instruction. Importantly, although the initial financial investment may appear substantial, long-term benefits include a reduction in the number of failed cannulation attempts, fewer procedure-related complications, improved time efficiency, and better staff and patient satisfaction. These gains have the potential to offset initial costs and generate cost-effectiveness at the system level, particularly in high-demand pediatric units.

While this review focused exclusively on ultrasound-guided techniques, it is important to note that other adjunctive technologies have also been explored for peripheral vascular access in pediatric populations. Near-infrared (NIR) imaging [46], for example, has been used to enhance the visualization of superficial veins, particularly in older children, by projecting real-time images of subcutaneous vasculature onto the skin. However, its effectiveness in infants is more limited due to the depth and small caliber of veins in this age group. Moreover, unlike ultrasound, NIR imaging does not allow for dynamic needle guidance during cannulation. As such, while NIR may serve as a complementary tool in selected cases, ultrasound remains the most comprehensive modality for both vein localization and real-time procedural assistance in infants. Future reviews may consider comparative analyses of these technologies to further guide evidence-based practice.

### 4.6. Long-Term Vascular Impact

Another important aspect that remains insufficiently addressed in the current literature is the potential long-term impact of repeated ultrasound-guided peripheral vascular cannulations on vascular health in infants. None of the studies included in this review reported follow-up data regarding vessel integrity, patency, or scarring beyond the immediate procedural outcomes. Given the anatomical fragility of infant veins and the need for frequent access in certain clinical scenarios, such as chronic conditions or prolonged hospital stays, future research should explore whether repeated ultrasound-guided peripheral vascular procedures may have cumulative effects on vascular structures. Understanding these long-term consequences is essential to ensure that the short-term benefits of improved cannulation success do not compromise the long-term viability of peripheral access sites.

### 4.7. Limitations

This review supports the efficacy and safety of ultrasound-guided peripheral vascular cannulation in pediatric patients, but several limitations must be acknowledged.

A major limitation across the included studies is the substantial clinical and methodological heterogeneity. Key contextual and procedural factors—such as operator experience, healthcare setting (academic vs. community), ultrasound equipment quality, use of anesthesia, and inclusion of simulated participants—were often inconsistently reported. Combined with small sample sizes, varied patient populations, and the incomplete documentation of clinical variables like age, sedation status, or underlying conditions, this variability hampers both data synthesis and the development of standardized, evidence-based protocols.

In terms of this review’s scope, the exclusion of non-open access studies, non-English/Spanish/Portuguese publications, and gray literature may have introduced selection bias. Moreover, variability in clinical settings—ranging from PICUs to emergency departments and neonatal units—raises concerns about the generalizability of findings due to differences in infrastructure, staffing, and institutional practices.

Additionally, the lack of randomization, blinding, and standardized outcome measures (e.g., pain, stress, and procedure time) across studies precluded meta-analysis.

To strengthen future evidence, well-designed, multicenter randomized trials with clear methodologies and standardized variables are needed. Studies should include multivariate adjustments and report on broader outcomes such as cost-effectiveness, hospital stay, patient and caregiver satisfaction, long-term vascular health, and the retention of ultrasound skills. These efforts will improve the reliability, applicability, and implementation of this technique in pediatric care.

## 5. Conclusions

The evidence synthesized in this systematic review consistently supports the use of ultrasound-guided peripheral vascular cannulation as a more effective, safer, and more patient-centered technique compared to the conventional or “blind” method, particularly in the infant population. Most of the included studies agree that ultrasound significantly increases first-attempt success rates, reduces the number of required punctures, and helps lower the stress associated with the procedure for both patients and their families—factors that are especially relevant in pediatric care.

## Figures and Tables

**Table 1:** Descriptive and methodological characteristics of the studies.

Authors	Country	Study Type	Setting	Sample
Castillo and Corral [35]	Spain	Prospective observational study	Resuscitation Unit, Gregorio Marañón Hospital, Madrid	107 arterial cannulations (new or replacement)
Takeshita et al. [36]	Japan	Randomized controlled trial	Single center, Osaka Women and Children’s Hospital	60 pediatric patients under 2 years requiring peripheral vascular catheterization in PICU
Oulego-Erroz et al. [37]	Spain	Prospective multicenter study	18 PICUs across Spain over a 6-month period	161 procedures in 128 patients, with a median age of 11 months and a median weight of 10 kg
Kleidon et al. [38]	Australia	Open-label, pragmatic, superiority, randomized trial	Queensland Children’s Hospital	164 children, median age 24 (10–120) months; 85 ultrasound-guided, 80 conventional cannulations
Triffterer et al. [39]	Austria	Prospective observational study	Surgical block	90 infants ≤ 12 months undergoing elective surgery with no visible veins needing great saphenous vein cannulation; two groups (0–6 months and 7–12 months)
Gopalasingam et al. [40]	Denmark	Randomized, controlled, crossover study	Surgical block	50 pediatric patients under 4 years under anesthesia undergoing low-risk elective procedures
Carrie Ng et al. [30]	United Kingdom	Cross-sectional study	Various pediatric emergency departments	18 nursing professionals—17 from pediatric emergency care—were analyzed
López-Álvarez et al. [31]	Spain	Descriptive observational study	Pediatric Intensive Care Unit, Maternal and Child University Hospital of the Canary Islands	25 healthcare professionals (56% physicians, 44% nurses)
McKinney et al. [32]	United States	Training program	NICU	12 NICU nurses
Hackett et al. [33]	United States	Training program	ICU of a quaternary urban university hospital	Eight nurses: six from PICU and two from the pediatric transport team
Andersen et al. [41]	—	Pilot study designed as a randomized controlled trial	University of Southern Denmark	19 students

**Table 2:** Study results grouped by themes.

Theme	Study	Results/Conclusions
Stress reduction	Castillo and Corral [35]	No statistically significant differences were found between using and not using the ultrasound-guided technique; pain was mostly associated with the use of anesthetics.
Reduction in number of punctures	Takeshita et al. [36]	Dynamic ultrasound-guided cannulation reduced the number of punctures and time used compared to static guidance (success: 86.7% vs. 60%; time: 51.5″ vs. 71.5″).
	Oulego-Erroz et al. [37]	Significant difference in overall success rate (83.7% vs. 62.7%) and complication reduction (10.8% vs. 32.5%) in cannulations performed by staff with less than 5 years of PICU experience.
	Kleidon et al. [38]	Success rate with ultrasound-guided cannulation was 85.7% (72 children) vs. 32.5% (26 children) with the conventional technique.
	Triffterer et al. [39]	Success rate by age group: 0–6 months = 96%, 7–12 months = 100%. Overall success rate: 98%.
	Gopalasingam et al. [40]	First-attempt success (ultrasound: 42/50 vs. conventional: 30/50; *p* = 0.029); overall success (50/50 vs. 42/50; *p* = 0.008). Number of punctures (60 vs. 84; *p* = 0.013); redirections (14 vs. 131; *p* < 0.001); longer distance from flexion fold (*p* < 0.001). No significant differences in the number of catheters used or total time. Needle handling time was longer in the ultrasound group (*p* = 0.011). Lower success rate in the palpation group with less visible/palpable veins (*p* = 0.006).
Nursing staff perception	Carrie Ng et al. [30]	The cannulation techniques were well received. In total, 82% performed at least one. The self-guided technique was favored by two professionals (65%) and continued to be used 3 months later (65%).
	López-Álvarez et al. [31]	Success rate: 79.7%; average attempts: 1.8 ± 1.2 globally, 1.49 ± 0.86 for ultrasound-guided; average time: 115.6 s ± 114.9 s; time to successful ultrasound-guided cannulation: 87.69 s ± 82.81 s. Reliability questionnaire scored 87.2%, with perceived utility rated highest (92.85%).
Training and education	McKinney et al. [32]	The program helped improve NICU nursing staff training.
	Hackett et al. [33]	The program increased PICU nursing staff success rate by 82.5%.
	Andersen et al. [41]	Success rate was 73% (22 out of 30 cannulations) in the virtual reality group vs. 22% (6 out of 27) in the group without virtual reality.

## Data Availability

The original contributions presented in this study are included in the article. Further inquiries can be directed to the corresponding author.
